# Temporal and spatial variation of extreme temperatures in an agro-pastoral ecotone of northern China from 1960 to 2016

**DOI:** 10.1038/s41598-018-27066-0

**Published:** 2018-06-08

**Authors:** Xuyang Wang, Yuqiang Li, Yinping Chen, Jie Lian, Yongqing Luo, Yayi Niu, Xiangwen Gong, Peidong Yu

**Affiliations:** 1 Northwest Institute of Eco-Environment and Resources, Chinese Academy of Sciences, Lanzhou, 730000 China; 2 University of Chinese Academy of Sciences, Beijing, 100049 China; 3 Naiman Desertification Research Station, Northwest Institute of Eco-Environment and Resources, Chinese Academy of Sciences, Tongliao, 028300 China; 4 School of Environmental and Municipal Engineering, Lanzhou Jiaotong University, Lanzhou, 730070 China

## Abstract

The agro-pastoral ecotone of northern China is one of the areas most sensitive to global temperature change. To analyze the temporal and spatial trends of extreme temperature events in this area, we calculated the values of 16 extreme-temperature indices from 1960 to 2016 based on data from 45 national meteorological stations. We found that the coldest-temperature indices decreased significantly and the warmest-temperature indices increased significantly. The warming of night temperatures contributed more than warming of day temperatures to the overall warming trend. In addition, the warm-temperature indices appeared to be increasing since the late 1980s and early 1990s. Overall, though the four extremal indices showed an increasing trend, the rate of change in the minimum temperature was greater than that of the maximum temperature; thus, the minimum temperature contributed most strongly to the overall temperature increases. The growing season is being prolonged in higher-elevation areas, but vegetation maturation in lower-elevation areas has been accelerated by the high temperatures, potentially leading to a shorter growing season at low altitudes. However, the impacts of land-use changes caused by human activities on the temperature increases will require additional study.

## Introduction

Climate change and human land-use activities are interacting strongly, in unprecedented ways, with rapid changes in living conditions and in the structure and function of ecosystems^1^. According to the third assessment report by IPCC, the global average temperature has risen by 0.6 ± 0.2 °C over the past 100 years, with the temperature in China rising slightly more (by 0.5 to 0.8 °C) than the global rate^2^. Simultaneously, extreme climate events have become more and more frequent, with heat waves, storms, outbreaks of pests, and other disasters caused by extreme weather becoming commonplace in recent years^3^. These events have had huge impacts on the world’s ecosystems and social systems. Therefore, researchers have increasingly focused on how to deal with the impacts of climate change, and especially extreme climate events, on ecosystems and human livelihoods, with the goal of promoting sustainable development of society and ecosystems^4–10^.

In China, both the frequency and the intensity of extreme climate events have changed significantly since the 1960s, but the trends have differed among regions^11^. Since the northern agro-pastoral ecotone, a key area for China’s food security, is situated in a transition zone between areas with semi-arid and arid climates, this region is very sensitive to climate change. The transition zone includes lands that have undergone erratic but often unsustainable land use due to decades of alternations and upheavals caused by agriculture and animal husbandry. Owing to climate warming, regional drying, and increasing population pressure, the cultivated area has gradually expanded and the pastoral areas have shrunk. Variations of land use patterns and the intensity of the land uses would change the hydrologic and thermal balances of the land in this region^12^. For example, land-use changes are primary driving forces for changes in the near-surface climate^13^. In addition, these changes will profoundly affect disease incidence, such as the spread of malaria in tropical Africa, beginning well before 2050^14^. In developed countries such as the United States, changes in landowner behavior have mitigated the effects of climate warming by increasing carbon sequestration^15^. Such changes have not yet occurred in China, despite large national-scale afforestation programs.

Northern China’s agro-pastoral ecotone plays an important role as an ecological barrier between the densely populated southern regions and the sparsely populated north, similar to that played by the Sahel zone in Africa. Changes in rates of global warming and in land-use patterns have complicated the trends and patterns of climate change in this region, thereby creating new challenges for the development of local agriculture and animal husbandry. In the context of increasingly severe impacts of extreme climate change on the ecosystems and residents of this area, developing a sustainability strategy requires us to first understand the nature of the changes, as these will determine the responses and adaptations required by human society and natural ecosystems. Obtaining this understanding requires a scientific assessment of the current pressure on the land system and its resilience against this pressure^16^. One way to achieve this goal will be to quantify the regional characteristics of the frequency and intensity of extreme climate events and their spatial and temporal trends. This knowledge will help us to develop an adaptive response strategy based on the pressures that social and ecological systems are facing and will face under climate change.

The objectives of the present study were to better understand the ecological fragility of northern China’s agro-pastoral ecotone in the modern context of climate change by (1) describing the temporal and spatial characteristics of extreme temperatures from 1960 to 2016, (2) analyzing the causes of differences in the temperature changes by comparing extreme-temperature indices among the regions of this area, and (3) using this data to improve our understanding of the complex interactions between the regional climate and global climate change.

## Results

### Temporal trends in the relative indices

**Figure 1:**
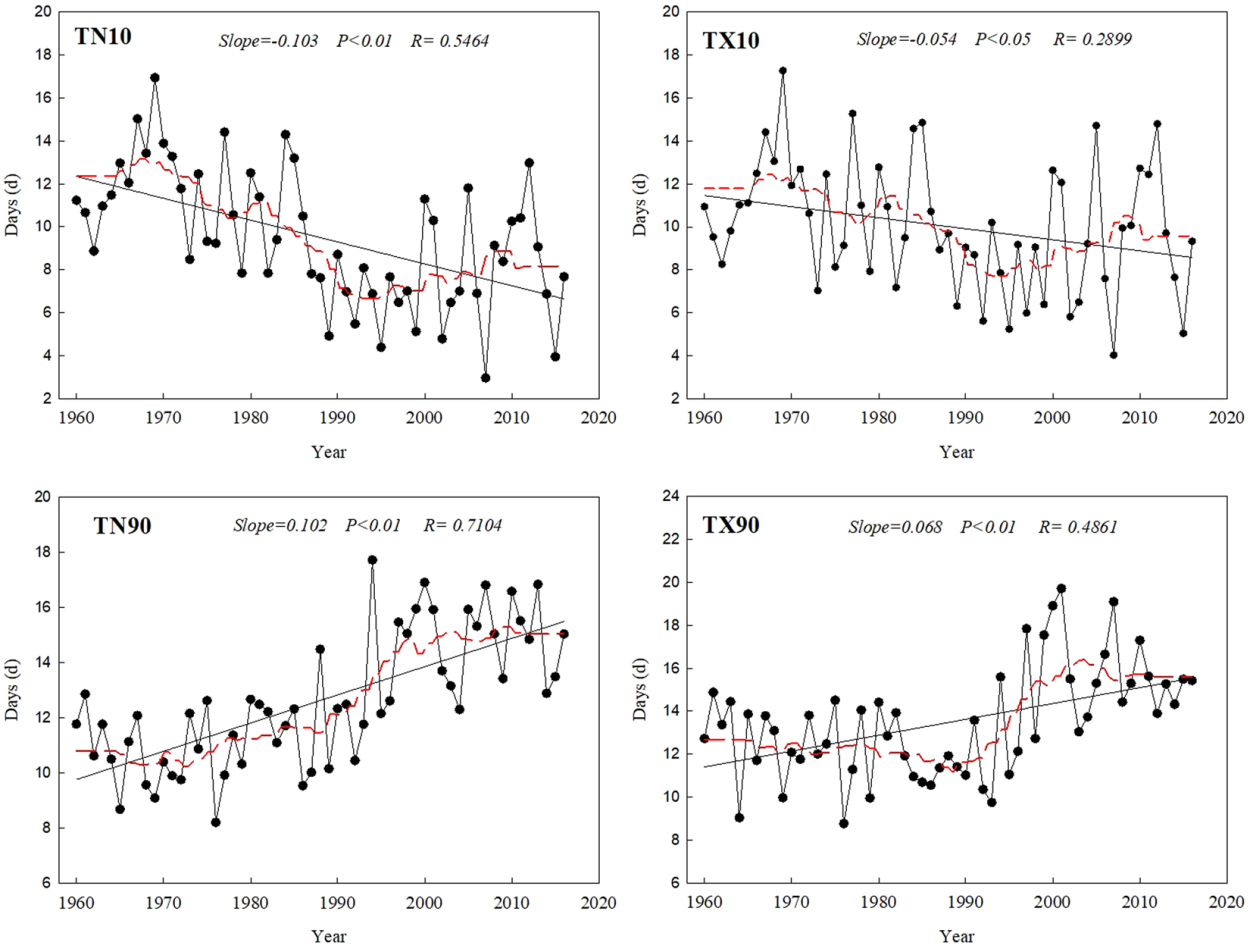
Long-term trends in the relative extreme-temperature indices: the numbers of cool nights (TN10), cool days (TX10), warm nights (TN90), and warm days (TX90). The indices are defined in Supplementary Table S1. Straight lines represent statistically significant linear regressions; the dashed red line is the smoothed 10-year running average. The sampling proportion = 0.18.

During the study period from 1960 to 2016, the average number of regional cool nights (TN10) and cool days (TX10) decreased significantly, at rates of 1.03 and 0.54 days/decade, respectively (Fig. 1). In contrast, the numbers of warm nights (TN90) and warm days (TX90) showed obvious increasing trends, with rates of 1.03 and 0.68 days per decade. In this study area, the mean number of cool nights and warm nights changed more obviously than the number of cool days and warm days. Both cool nights and cool days appeared to peak in 1969, with maximum values of about 17 days. The decrease in the number of cool nights was faster than the decrease in the number of cool days. The numbers of warm days and warm nights had similar trends, with fluctuations after a short decrease in the 1960s. The smoothed data (red lines, based on the 10-year running average, with a sampling proportion = 0.18) for TN10 and TX10 showed a decreasing trend, with fluctuations, but with a predominantly upward trend after the 1990s (Fig. 1). The smoothed data for TN90 and TX90 began to exhibit a dramatic upward trend in the late 1980s and early 1990s, but TN90 increased more rapidly than TX90.

### Spatial trends in the relative indices

**Figure 2:**
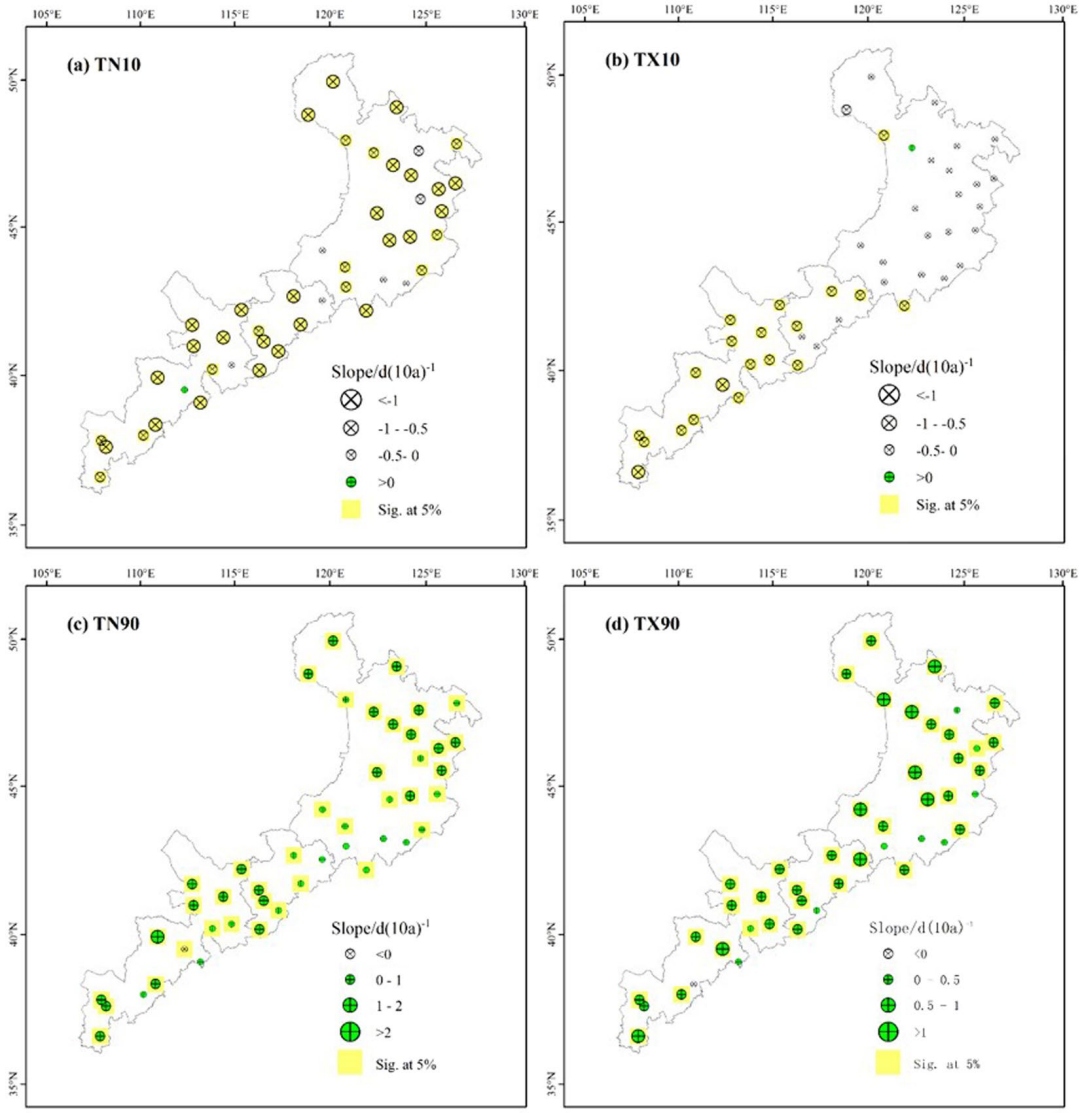
Spatial distribution of the relative extreme-temperature indices: the numbers of (**a**) cool nights (TN10), (**b**) cool days (TX10), (**c**) warm nights (TN90), and (**d**) warm days (TX90). The descriptions of the indices are shown in Supplementary Table S1. Increasing trends are shown as the green symbols, decreasing trends as white symbols. The station points with the background of yellow color represent significance (*F* test, *P* < 0.05).

For the number of cool nights (TN10), all stations except one station in the western part of the study area showed a decreasing trend, and the trend was significant (*P* < 0.05) for 82% of the stations (Fig. 2). A significantly decreasing trend in the number of cool days was observed at most stations in the western and central regions, with decreases ranging from 1.24 to 0.40 days per decade, whereas stations in the eastern region decreased by 0.60 to 0.18 days per decade. However, only two of the stations in the eastern region had statistically significant changes. The rate of change in the number of cool days decreased from west to east. For the warm nights and warm days, almost 87 and 80% of the stations, respectively, showed increasing trends, and the rate of change in the number of warm nights was obviously higher than that for the number of warm days. Overall, from west to east, the rate of change in the number of warm nights decreased to a minimum near the central part of the study area, and then increased. In addition, greater rates of increase in the number of warm days were observed in the eastern part, with rates of more than 1 °C per decade.

### Temporal trends in the absolute indices

**Figure 3:**
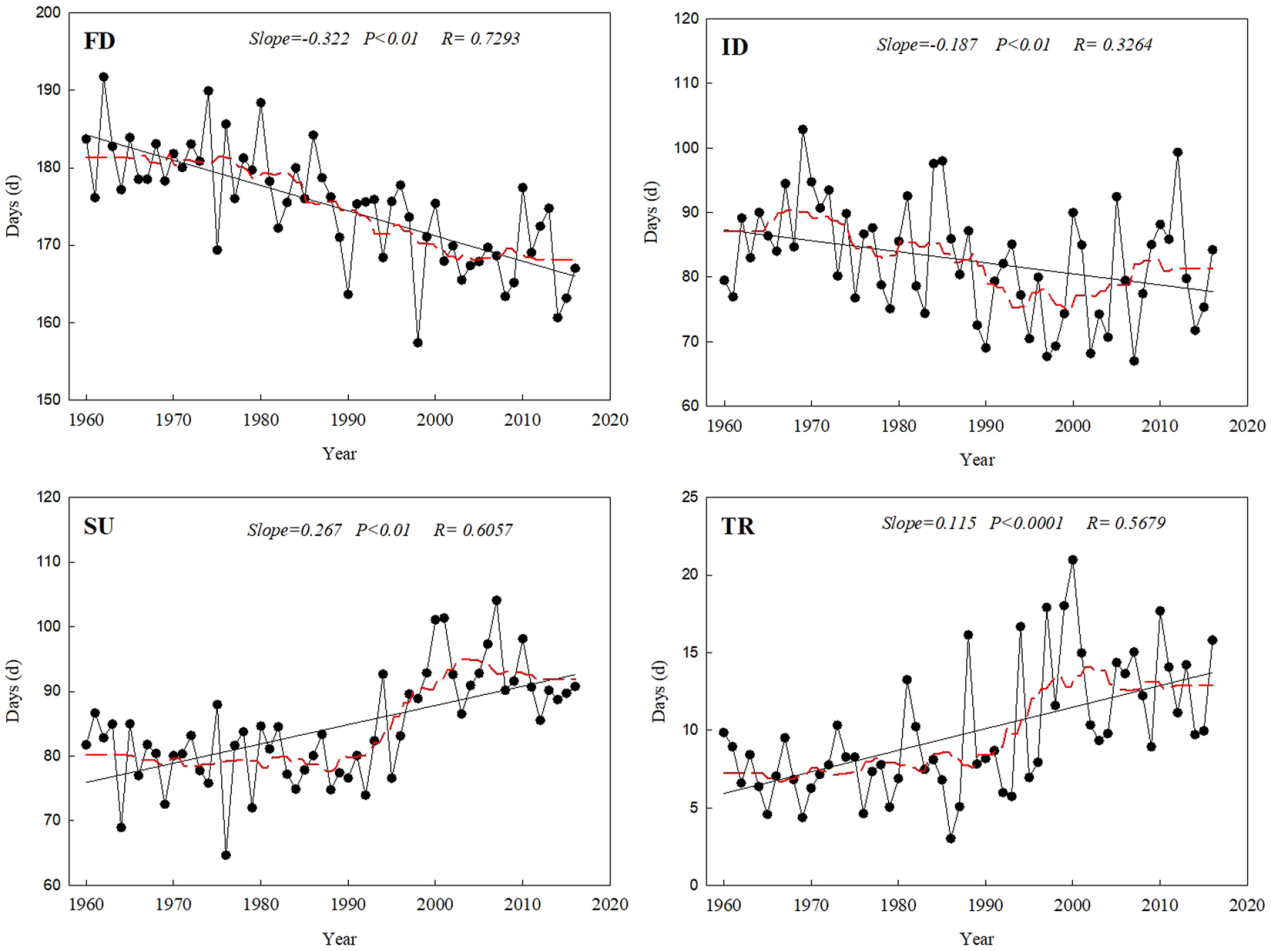
Long-term trends in the absolute extreme-temperature indices: the numbers of frost days (FD), ice days (ID), summer days (SU), and tropical nights (TR). The descriptions of the indices are shown in Supplementary Table S1. Straight lines represent statistically significant linear regressions and the dashed red line is the smoothed 10-year running average. The sampling proportion = 0.18.

The absolute indices (Fig. 3) showed trends similar to those of the relative indices. The warm indices increased significantly and the cool indices decreased significantly. The mean annual number of days decreased by 3.22 per decade (*P* < 0.001) for frost days (FD), and by 1.87 per decade (*P* < 0.05) for ice days (ID), but the number of ice days began to increase in the early 1990s. In contrast to the cool indices, the numbers of summer days (SU) and tropical nights (TR) increased rapidly, at rates of 2.67 and 1.15 days per decade (*P* < 0.001), respectively, with a particularly sharp increase since 1990.

### Spatial trends in the absolute indices

**Figure 4:**
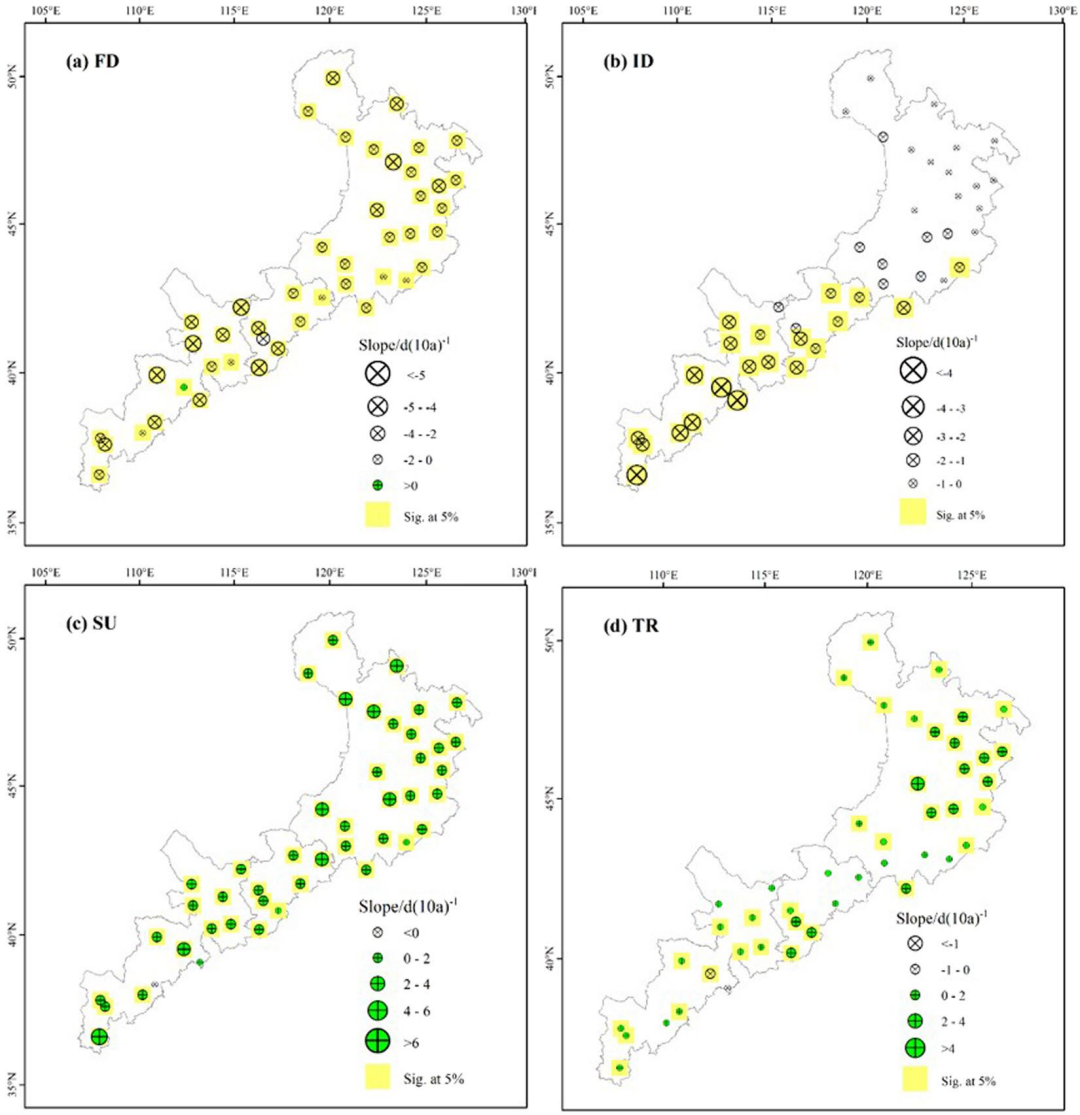
Spatial distribution of the absolute extreme-temperature indices: the numbers of (**a**) frost days (FD), (**b**) ice days (ID), (**c**) summer days (SU), and (**d**) tropical nights (TR). The descriptions of the indices are shown in Supplementary Table S1. Increasing trends are shown as the green symbols, decreasing trends as white symbols. The station points with the background of yellow color represent significance (*F* test, *P* < 0.05).

The numbers of frost days and cool nights showed similar spatial trends, with increasing trends at the same sites in the western region (Fig. 4). In addition, for the number of frost days, most other stations showed decreases ranging from 0 to 5 days per decade. Furthermore, the number of ice days showed negative trends at all meteorological stations, but most of the entire eastern stations didn’t show a significant trend. The rate of change in the number of ice days decreased from the western part of the study area to the eastern part, at rates of 2.93 and 0.96 days per decade, respectively. For the number of summer days, most of the stations showed obvious increasing trends, and 98% of the stations had a significant trend (*P* < 0.05). For the number of tropical nights, 78% of the stations showed a significant increasing trend, and the rate of change in the number of tropical nights increased from the western part of the study area to the eastern part, at average rates of 0.71 and 1.81 days per decade, respectively.

### Temporal trends in the duration indices

**Figure 5:**
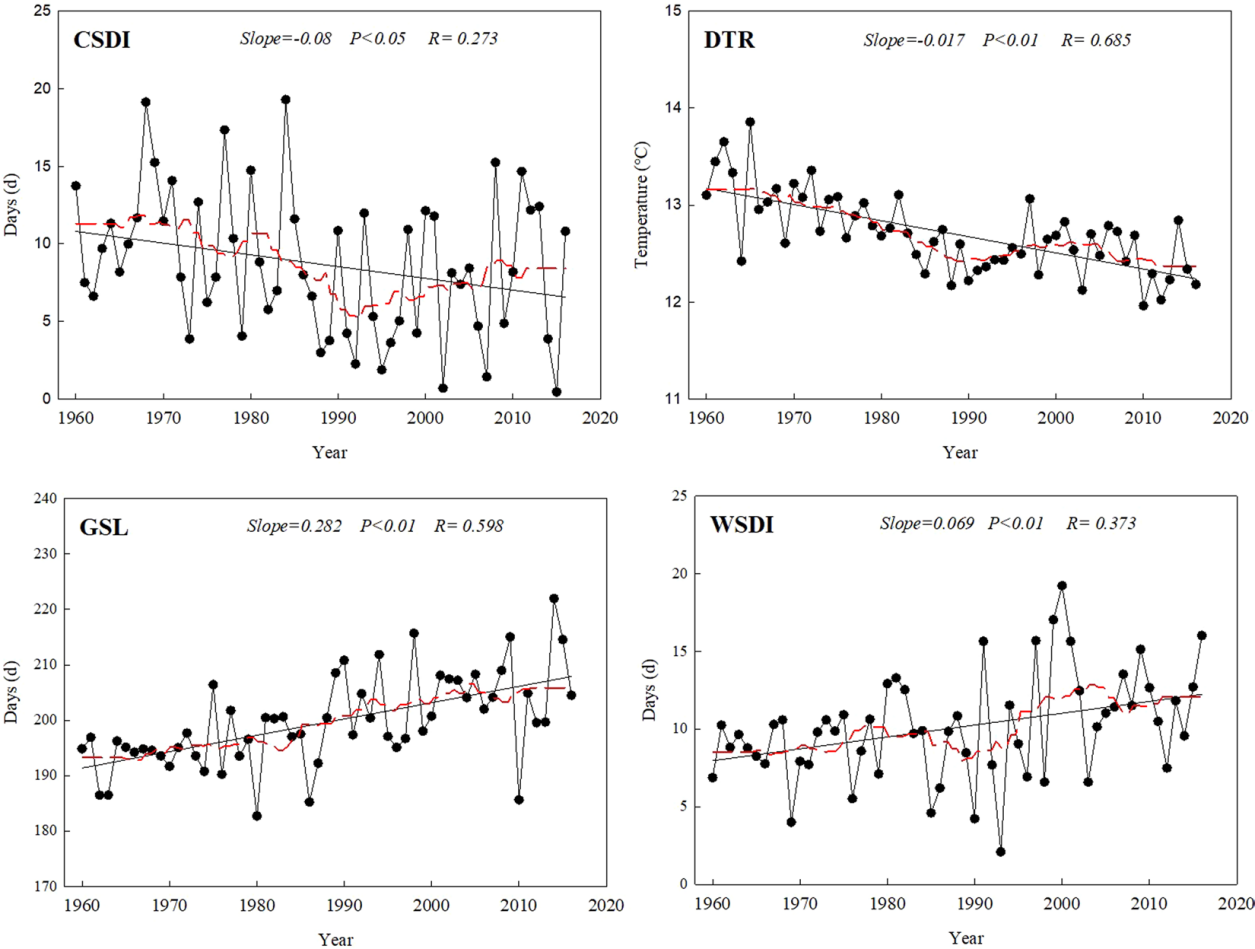
Long-term trends in the duration extreme-temperature indices: cold spell duration (CSDI), diurnal temperature range (DTR), growing season length (GSL), and warm spell duration (WSDI). The descriptions of the indices are shown in Supplementary Table S1. Straight lines represent statistically significant linear regressions and the dashed red line is the smoothed 10-year running average. The sampling proportion = 0.18.

Both the average cold spell duration (CSDI) and the diurnal temperature range (DTR) showed significant decreasing trends, at rates of 0.80 days per decade and 0.17 °C per decade, respectively (Fig. 5). However, the warm spell duration (WSDI) and growing season length (GSL) increased significantly, at rates of 2.82 and 0.69 days per decade. Based on the running average, CSDI and DTR reached their minima around 1992. Both GSL and WSDI showed fluctuation despite the overall increasing trend, but WSDI fluctuated more widely than GSL, especially after 1990, and the rate of increase of WSDI was slower than that of GSL.

### Spatial trends in the duration indices

**Figure 6:**
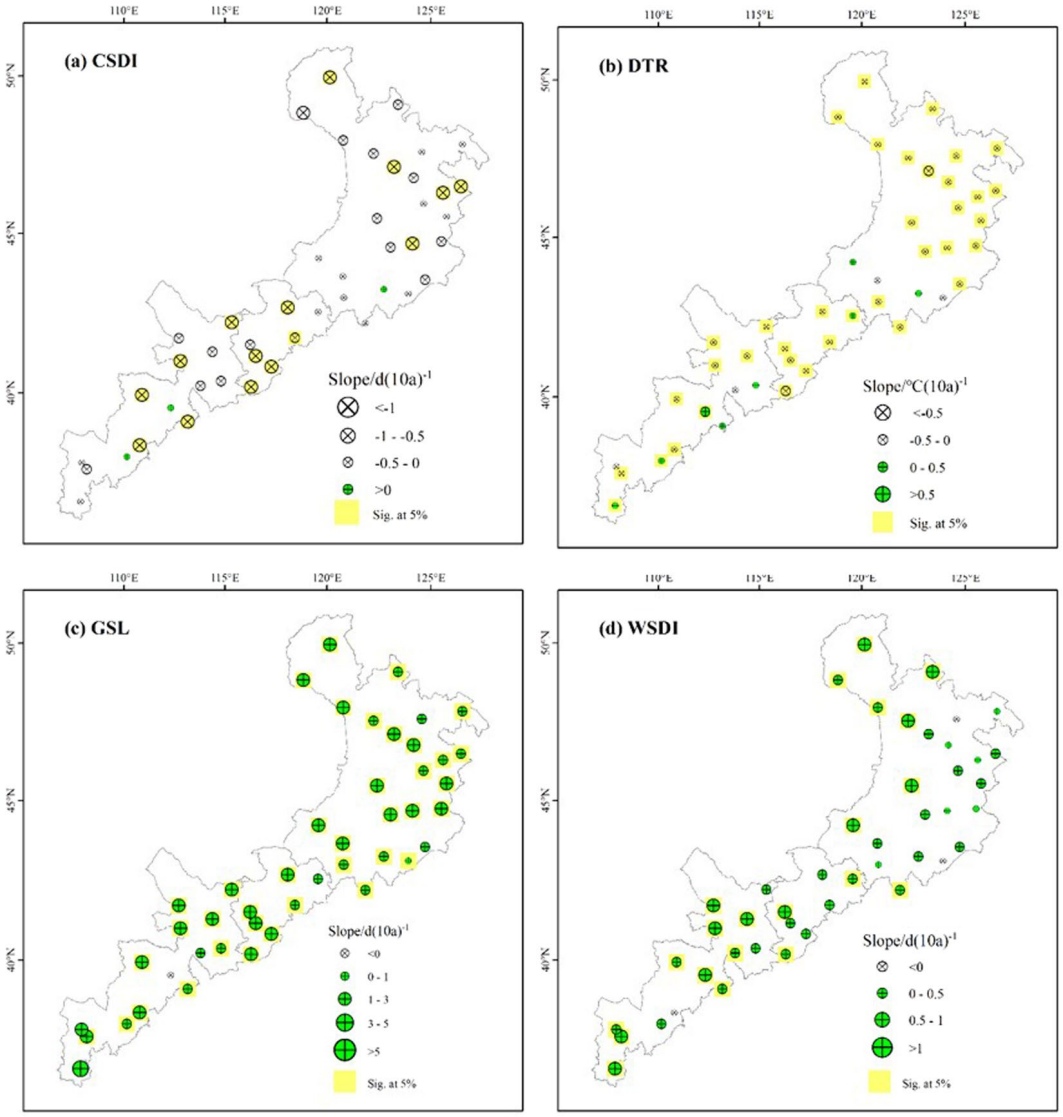
Spatial distribution of the extreme-temperature duration indices: (**a**) cold spell duration (CSDI), (**b**) diurnal temperature range (DTR), (**c**) growing season length (GSL) and (**d**) warm spell duration (WSDI). The descriptions of the indices are shown in Supplementary Table S1. Increasing trends are shown as the green symbols, decreasing trends as white symbols. The station points with the background of yellow color represent significance (*F* test, *P* < 0.05).

Although most of the stations showed decreasing trends in CSDI, this trend was only significant for 36% of the stations (Fig. 6). From the western part of the study area to the eastern part, the average rate of decrease in the cold spell duration changed from 0.76 to 1.03 days per decade in the central region, and decreased to an average of 0.68 days per decade in the eastern region. For DTR, most of the stations showed little difference, with most rates of decrease between 0.5 and 0 °C per decade. Nearly 36% of the stations showed significant decreasing trends, but 18% of the stations showed significant increasing trends. In contrast, both WSDI and GSL mainly showed positive trends. In general, the rates of increase in GSL were high, with about 82% of stations increasing by more than 2 days per decade. The Huan County site had the highest rate of change (5.67 days per decade), and was in the western region. Overall, WSDI increased more slowly than GSL. For WSDI, the rate of increase ranged from 0.07 to 1.72 days per decade. Most stations with a significantly increasing trend were located in the western part of the study area.

### Temporal trends in the extremal indices

**Figure 7:**
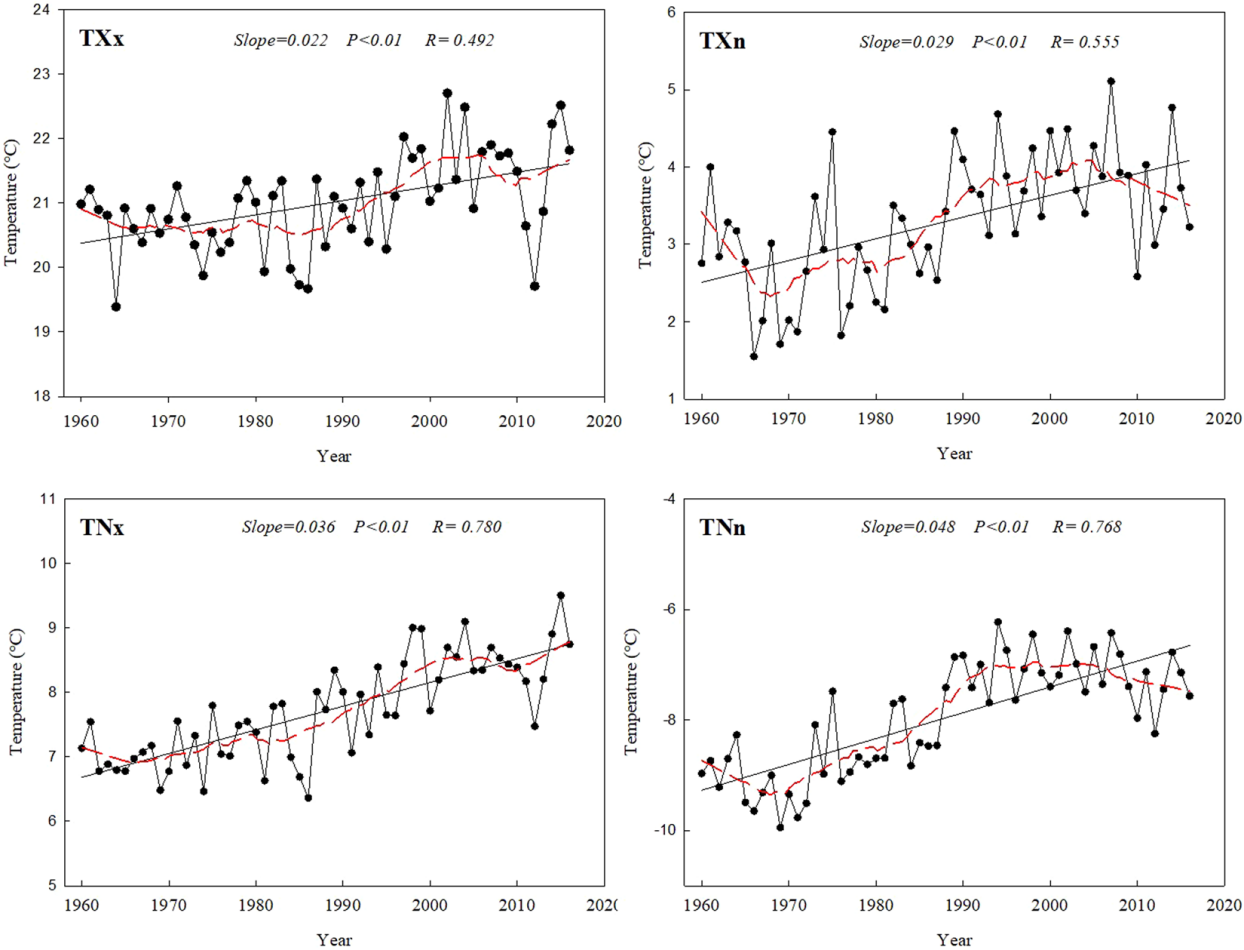
Long-term trends in the extremal temperature indices: maximum value of the daily maximum temperature (TXx), minimum value of the daily maximum temperature (TXn), maximum value of the daily minimum temperature (TNx), and minimum value of the daily minimum temperature (TNn). The descriptions of the indices are shown in Supplementary Table S1. Straight lines represent statistically significant linear regressions and the dashed red line is the smoothed 10-year running average. The sampling proportion = 0.18.

All four of the extremal indices showed slow but significant increasing trends (Fig. 7). The rate of increase for the maximum value of the daily maximum temperature (TXx), minimum value of daily maximum temperature (TXn), maximum value of daily minimum temperature (TNx), and minimum value of daily minimum temperature (TNn) increased at rates of 0.22, 0.29, 0.36, and 0.48 °C per decade, respectively. Based on the smoothed averages, the four extremal indices displayed the same trend: they decreased during the first decade of the study period and then increased continuously. However, TXn and TNn appeared to show decreasing trends after 2000.

### Spatial trends in the extremal indices

**Figure 8:**
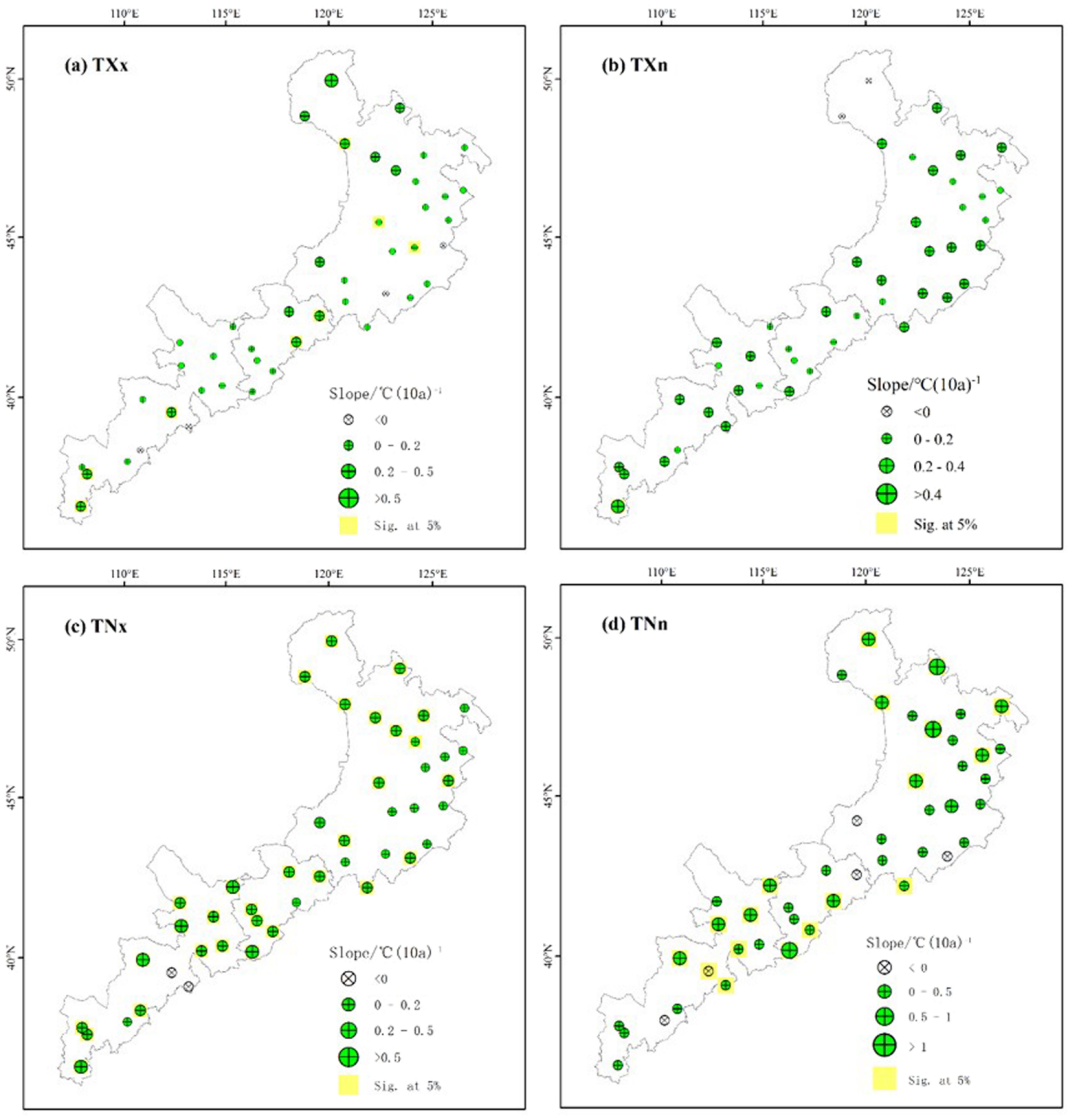
Spatial distribution of the extremal temperature indices: (**a**) maximum value of the daily maximum temperature (TXx), (**b**) minimum value of the daily maximum temperature (TXn), (**c**) maximum value of the daily minimum temperature (TNx), and (**d**) minimum value of the daily minimum temperature (TNn). The descriptions of the indices are shown in Supplementary Table S1. Increasing trends are shown as the green symbols, decreasing trends as white symbols. The station points with the background of yellow color represent significance (*F* test, *P* < 0.05).

The spatial distribution of the four extremal indices showed similar trends (Fig. 8). For TXx, almost all stations showed an increasing trend, with 18% of the stations showing statistically significant trends (*P* < 0.05). Almost 73% of the stations had rates of increase between 0 and 0.2 °C per decade, but only one station had a rate >0.5 °C per decade. For TXn, nearly all stations showed an increasing trend, except for the two easternmost stations (which showed decreases); however, only one station in the southernmost part of the study area showed a significant trend. All stations in the central and eastern parts of the study area showed increasing trends in TNx, but two stations showed the opposite trend in the western part of the study area. In addition, almost 58% of stations showed significant increasing trends (*P* < 0.05). Overall, TNn increased relatively rapidly, at up to 1.3 °C per decade. Approximately 33% of the stations showed significant increasing trends, and these stations were mainly located in the central and eastern parts of the study area.

### Regional differences in the extreme-temperature indices

To compare the extreme-temperature indices and their rates of change in different regions, we divided the study area into three zones (the western, central, and eastern regions; Table 1). For the absolute indices, the annual average number increased continuously from west to east; However, the rate of change of ID decreased significantly (one-way ANOVA followed by LSD test, *P* < 0.01) in the following order: western region (2.93) > central region (1.86) > eastern region (0.96). The values of the relative indices ranged from 8.93 to 13.76 days in different regions. For the relative cool indices, TX10 increased significantly from the western region to the eastern region, and TN10 was lowest in the central region (though the difference was not significant), but the relative warm indices showed the opposite trend. The rate of decrease of TX10 became significantly lower moving from the western region to the eastern region, and both TN10 and TX90 showed their maximum value in central region (though the differences were not significant). For the duration indices, the maximum durations of WSDI and CSDI appeared in the western and eastern regions, respectively, although the difference was only significant for CSDI, whereas DTR and GSL decreased from the western region to the eastern region, although the difference was only significant for DTR. The rate of change of TNn was lowest in the central region, though the difference was not significant, and although the remaining three extremal indices all decreased from the western region to the eastern region, the differences were not significant.

**Table 1:** Differences in the means and rates of change of the extreme-temperature indices for the three regions of the agro-pastoral ecotone.

Indices	Western region (*n* = 14)		Central region (*n* = 7)		Eastern region (*n* = 24)	
	Average	Rate of change	Average	Rate of change	Average	Rate of change
FD	167.62 ± 5.09	−3.32 ± 0.58	171.90 ± 8.22	−3.78 ± 0.67	177.87 ± 3.87	−3.19 ± 0.19
SU	80.21 ± 6.14	2.77 ± 0.37	82.00 ± 11.67	2.89 ± 0.28	85.10 ± 4.05	3.13 ± 0.15
ID	61.10 ± 6.35**B**	−2.93 ± 0.23**a**	71.20 ± 8.40**B**	−1.86 ± 0.20**b**	96.96 ± 4.69**A**	−0.96 ± 0.10**c**
TR	4.23 ± 1.00**B**	0.71 ± 0.27**b**	7.58 ± 3.09**A**	1.33 ± 0.51**ab**	13.44 ± 1.66**A**	1.81 ± 0.24**a**
TX10	9.55 ± 0.08**B**	−0.79 ± 0.05**a**	9.69 ± 0.09**B**	−0.57 ± 0.06**b**	10.16 ± 0.05**A**	−0.34 ± 0.31**c**
TN10	9.51 ± 0.17	−0.98 ± 0.16	8.93 ± 0.28	−1.36 ± 0.21	9.45 ± 0.14	−0.94 ± 0.08
TX90	13.33 ± 0.12**B**	0.69 ± 0.10	13.76 ± 0.19**A**	0.85 ± 0.10	13.12 ± 0.09**B**	0.73 ± 0.06
TN90	12.70 ± 0.29	1.08 ± 0.23	12.63 ± 0.41	0.97 ± 0.20	12.21 ± 0.11	1.01 ± 0.09
WSDI	10.14 ± 0.22	0.93 ± 0.12	9.58 ± 0.18	0.85 ± 0.08	9.94 ± 0.14	0.65 ± 0.10
CSDI	8.55 ± 025**A**	−0.76 ± 0.18	7.43 ± 0.42**B**	−1.03 ± 0.21	8.90 ± 0.14**A**	−0.68 ± 0.10
DTR	13.22 ± 0.32**A**	−0.06 ± 0.08	13.00 ± 0.34**A**	−0.22 ± 0.08	12.32 ± 0.16**B**	−0.20 ± 0.03
GSL	203.22 ± 4.33	3.06 ± 0.42	199.67 ± 6.61	3.18 ± 0.41	191.07 ± 3.47	2.82 ± 0.16
TNn	−7.42 ± 0.73	0.32 ± 0.10	−7.28 ± 1.35	0.39 ± 0.17	−8.44 ± 0.70	0.38 ± 0.06
TXn	4.47 ± 0.73	0.24 ± 0.02	4.12 ± 1.20	0.15 ± 0.04	2.41 ± 0.59	0.22 ± 0.03
TXx	21.60 ± 0.51	0.13 ± 0.03	21.28 ± 0.80	0.23 ± 0.06	20.58 ± 0.47	0.16 ± 0.03
TNx	8.16 ± 0.49	0.34 ± 0.06	7.91 ± 0.81	0.33 ± 0.06	7.41 ± 0.51	0.25 ± 0.03

Note: For a given parameter, values labeled with different letters differ significantly (*P* < 0.05; one-way ANOVA, followed by LSD test).

### Correlations among the extreme-temperature indices

As shown in the correlation matrix (Table 2), there were significant correlations between many pairs of indices. For the absolute indices, we found significant positive correlations between the warm indices and between the cold indices. In contrast, each warm index was negatively correlated with each cold index. This is consistent with the abovementioned results, in which the cold indices showed significantly decreasing trends, whereas the warm indices showed significantly increasing trends. In addition, we found significant negative correlations between the cool absolute indices and the extremal indices, but significant positive correlations between the warm absolute indices and the extremal indices. TR was positively correlated with GSL, but significantly negatively correlated with DTR. GSL was significantly negatively correlated with FD and ID, but significantly positively correlated with SU and TR.

**Table 2:** The correlation matrix for the relationships among the extreme-temperature indices in the agro-pastoral ecotone.

Indices	FD	SU	ID	TR	GSL	TX10	TX90	TN10	TN90	WSDI	CSDI	DTR	TNn	TXn	TXx	TNx
FD	1															
SU	−0.85^**^	1														
ID	0.84^**^	−0.65^**^	1													
TR	−0.56^**^	0.74^**^	−0.17	1												
GSL	−0.97^**^	0.86^**^	−0.92^**^	0.49^**^	1											
TX10	0.26	−0.06	0.45^**^	0.27	−0.30^*^	1										
TX90	0.22	−0.27	−0.05	−0.44^**^	−0.14	−0.19	1									
TN10	0.11	−0.08	−0.04	−0.11	−0.04	0.27	0.00	1								
TN90	0.07	−0.33^*^	0.07	−0.33^*^	−0.13	−0.17	0.11	−0.59^**^	1							
WSDI	0.06	−0.18	0.09	−0.31^*^	−0.11	−0.08	0.34^*^	0.08	0.27	1						
CSDI	0.06	0.04	0.18	0.10	−0.08	0.22	−0.26	0.71^**^	−0.47^**^	0.08	1					
DTR	0.25	−0.12	−0.27	−0.48^**^	−0.04	−0.24	0.38^*^	0.28	−0.19	−0.10	−0.06	1				
TNn	−0.98^**^	0.87^**^	−0.79^**^	0.59^**^	0.95^**^	−0.18	−0.16	−0.13	−0.11	−0.08	−0.09	−0.27	1			
TXn	−0.93^**^	0.87^**^	−0.92^**^	0.46^**^	0.98^**^	−0.32^*^	−0.09	−0.05	−0.17	−0.16	−0.13	0.08	0.93^**^	1		
TXx	−0.88^**^	0.87^**^	−0.88^**^	0.45^**^	0.94^**^	−0.13	−0.04	0.06	−0.24	−0.11	−0.04	0.13	0.89^**^	0.95^**^	1	
TNx	−0.97^**^	0.88^**^	−0.83^**^	0.63^**^	0.97^**^	−0.16	−0.21	−0.03	−0.18	−0.16	−0.04	−0.18	0.96^**^	0.93^**^	0.92^**^	1

Note: ** and * represent significance at P < 0.01 and P < 0.05, respectively. All other values are not significant.

## Discussion

**Figure 9:**
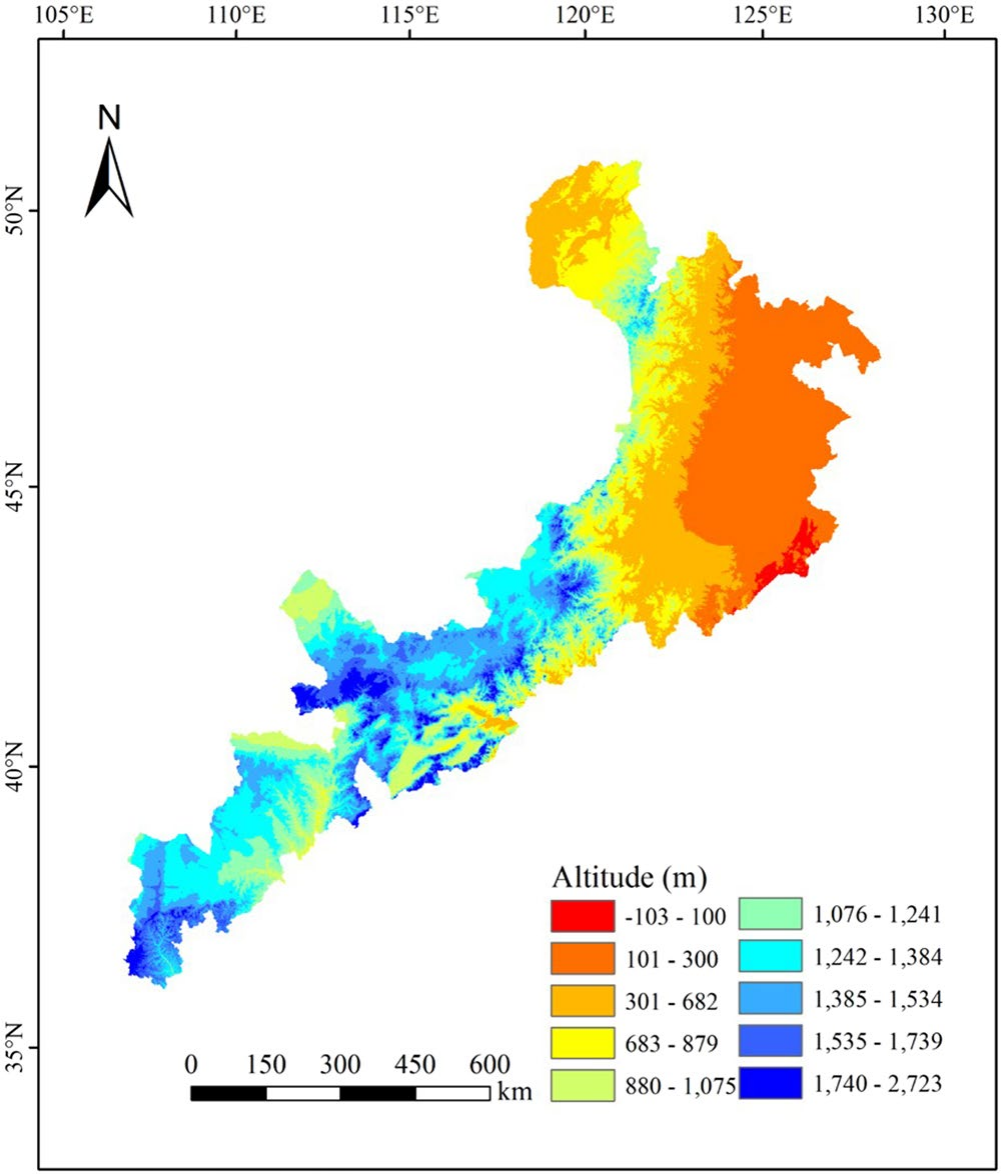
Topography (elevations) of the agro-pastoral ecotone in northern China. The dataset was provided by the Data Center for Resources and Environmental Sciences, Chinese Academy of Sciences (http://www.resdc.cn).

We found that the cool indices showed significant decreasing trends from 1960 to 2016, whereas the warm and extremal indices showed significant increasing trends. For example, the numbers of cool days and cool nights both decreased over time, whereas the numbers of warm days and warm nights increased. This is consistent with previous conclusions based on research in the Qilian Mountains^17^ and the Yangtze River Basin^18^ in China. The changes of TN10 and TN90, which characterized the night temperature, were greater than the changes in TX10 and TX90, which characterized the daytime temperature. This indicated that the warming of the night temperature contributed more than warming of day temperatures to the overall warming process. This conclusion has been confirmed by other scholars^19–21^. The numbers of cool days and cool nights were similar before the 1980s. However, the rate of decrease in the number of cool nights was greater than that in the number of cool days after the 1980s. The numbers of warm days and warm nights increased dramatically after 1990. The western Mu Us Sand land in the Ordos Plateau has an average elevation ranging from 1400 to 1500 m asl, and its eastern part belongs to the transition zone between the Northeast Plains and the Inner Mongolia Plateau, where the elevation ranges from 200 to 700 m^22^. Both SU and TR are warm indices, and their rates of increase increased from west to east with the gradual decline in elevation (Fig. 9). Due to the higher freedom degree of the air in the Northeast Plains, they showed greater warming than was recorded at stations on the Ordos Plateau^23^. Other researchers^24^ have studied the impact of topographic differences on the global temperature change and reached similar conclusions. Thus, the elevation differences and complicated topography of China’s agro-pastoral ecotone appear to have greatly influenced the changes in the extreme temperatures.

Though the four extremal indices all showed significant increasing trends, the rates of change of TNx and TNn were greater than those of TXx and TXn. This conclusion is similar to previous results from Heilongjiang Province^25^. Therefore, the change in the minimum temperatures appears to contribute more strongly to the overall increase of temperatures. DTR represents the monthly mean difference between TXx (the maximum temperature) and TNn (the minimum temperature), therefore most of the stations showed decreasing DTR, and the rate of increase of TNn (0.47 °C per decade) was greater than that of TXx (0.22 °C per decade). In addition, GSL (growing season length) decreased from 203.22 days in the west to 191.07 days in the east, although the difference was not significant. The mechanism by which climate change influences vegetation phenology is complicated. The length and changes of the vegetation growing season are closely related to climate change^26^. The elevation of our study area gradually decreases from west to east (Fig. 9). The growing season is prolonged by warming in areas with a higher elevation because cold temperatures are a severe constraint on growth, whereas vegetation maturation is accelerated by high temperatures at lower elevations, potentially leading to a shorter growing season in some low-elevation areas^27^. In addition, CSDI decreased by 0.76 days per decade, and WSDI increased by 0.77 days per decade. These results are similar to previous results for CSDI (a decrease of 0.67 days per decade) and WSDI (an increase of 0.83 days per decade) in China’s Yangtze River Basin^18^, but quite different from the results for CSDI (a decrease of 1.12 days per decade) and WSDI (and increase of 3.47 days per decade) in China’s Yellow River Basin^28^.

Various land use types have undergone great changes from 1980 to 2015 in the agro-pastoral ecotone (Table 3). Since the 1980s, the areas of cropland and urban land have expanded continuously. As a result, the areas of woodland and grassland have decreased. Because land use and cover change play important roles in radiative forcing, these changes will significantly affect the regional climate^29^. For example, deforestation, reclamation of grassland for agriculture, and deterioration of natural ecosystems could increase atmospheric CO_2_ concentrations by increasing CO_2_ emission or decreasing sequestration^30–32^, eventually leading to climate warming. Therefore, the observed changes in land-use patterns may partially explain the rising temperatures in this study area. In addition, the expanding area of urban land would strengthen the heat island effect, leading to elevated temperatures^33^. Atmospheric circulation is another important factor leading to rising temperatures. Due to the dominance of anticyclonic circulation over central China and Japan in the middle to low troposphere in summer and autumn^34^, the Asian monsoon system falls off ^35^. The climate of the agro-pastoral ecotone is controlled by inflows of dry warm air, while the strengthened north wind blocks flows of moisture of ocean flowing to our study area. Furthermore, in spring and winter, strengthening of the cyclonic system increases the strength of the west wind, which in turn weakens the winter monsoon and reduces cold air into the study area^18^, eventually leading to rapid temperature increases.

**Table 3:** Changes in the area of each land use in the agro-pastoral ecotone of northern China from 1980 to 2015.

Year	Cropland (km^2^)	Woodland (km^2^)	Grassland (km^2^)	Urban areas (km^2^)
1980	171006	90749	283609	11938
1990	173210	88795	281024	12684
1995	180529	91486	278106	13030
2000	185692	87863	272743	12850
2005	185435	88915	272212	13219
2010	185440	89067	272317	13411
2015	185680	88894	270896	15547

The agro-pastoral ecotone that we studied is a transitional zone between regions with a continental climate and regions with a monsoon climate, so climate change in this region is unstable, and the impact of land-use changes caused by human activities on regional temperature increases may be significant. Because of the importance of this region for China’s food security, it will be necessary to examine these impacts more carefully. To reduce some of the negative trends we observed in this study, researchers should study the beneficial impacts of returning cropland to forests and grassland, thereby improving protection of the ecological environment and increasing carbon sequestration.

## Data and Methods

### Study area

**Figure 10:**
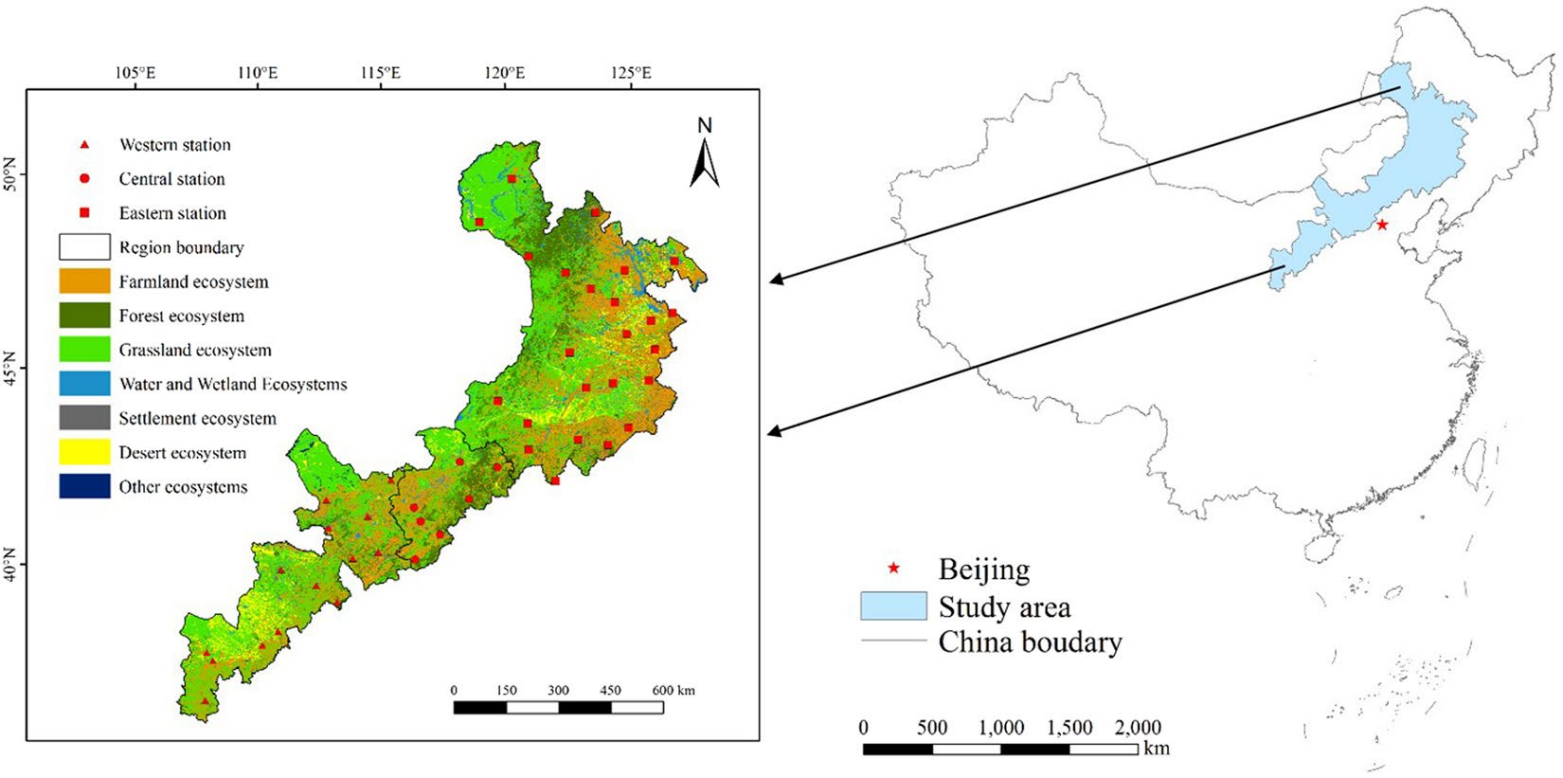
Location of the study area and the meteorological stations. The study area was divided into three zones (the western, central, and eastern regions), with 14, 7, and 24 meteorological stations, respectively. The figure also shows the distribution of ecosystems in the study area in 2015. The dataset was provided by the Data Center for Resources and Environmental Sciences, Chinese Academy of Sciences (http://www.resdc.cn).

We conducted our research in the agro-pastoral ecotone of northern China (Fig. 10), an area whose boundaries were defined based on field investigations by Zhao Halin *et al*.^22^ according to China’s climate zoning^36^, planting zoning^37^, desertification control zoning^37^, and other research results^38^. This region is distributed on both sides of the 400-mm precipitation isohyet, with annual precipitation of 300 to 400 mm on the northern and western sides of this line, and annual precipitation of 400 to 450 mm on the southern and eastern sides of the line. The ecotone covers a large area due to more than 10° north-south span of latitudes, so temperature and the length of the growing season vary widely. As a result, the annual average temperature ranges widely: from 3 to 7 °C in the Horqin Sandy Land in the east; between 0 and 1 °C in the middle part of the ecotone because of the high elevation; and between 6.0 and 9.0 °C in the Mu Us Sand Land in the west.

The topography also varies greatly within the study area. The northernmost part of the region includes the Hulunbeier Plateau, at an elevation of 650 to 750 m asl. Moving southward into the Horqin Sandy Land, a transitional zone between China’s Northeast Plains and the Inner Mongolia Plateau, the elevation ranges between 200 and 700 m asl. In the western Mu Us Sand Land, on the Ordos Plateau, the elevation ranges from 1400 to 1500 m asl (Fig. 9).

The study area represents a transitional zone between forest and grassland. The native vegetation before the modern human impacts was a sparse grassland. However, the vegetation in most of the study area has become seriously degraded by unsustainable human activities. Most of the natural vegetation has been replaced by plants capable of surviving in degraded sandy soils (i.e., psammophytes).

### Data sources

We obtained meteorological data, including daily maximum and minimum temperatures, from 45 meteorological stations distributed throughout the ecotone (Supplementary Table S1). The data were obtained from the shared database of the National Climate Center of the China Meteorological Administration (http://www.nmic.cn/en). Because of recent quality control efforts to improve the database, the quality and completeness of the data from 1960 to 2016 have improved greatly compared with similar products released in the past; the proportion of correct data is now believed to approach 100%.

### Quality control and homogeneity testing

To further ensure data quality, we performed several quality-control steps. First, we replaced all missing values (currently coded as −99.9) with an internal format that the R statistical software recognizes (i.e., NA, for not available), and replaced all unreasonable values into that category. Examples include a daily maximum temperature that was less than the daily minimum temperature, and vice versa. In addition, we identified outliers in the daily maximum and minimum temperatures. We defined outliers as values more or less than 3σ from the long-term mean value for that day. We then used the RHtestsV4 software (http://etccdi.pacificclimate.org/sofware.html) to detect multiple change points (shifts) that could exist in a data series that may have first-order autoregressive errors. This analysis was based on the penalized maximal *t* test^39^ and the penalized maximal *F* test^40^, which are embedded in a recursive testing algorithm^41^ that accounts empirically for a lag −1 autocorrelation (if any) in the time series.

### Extreme indices

We calculated the values of 16 extreme-temperature indices using the RClimDex software (http://etccdi.pacificclimate.org/software.shtml). The definitions of the extreme-temperature indices (Supplementary Table 2) are those recommended by the Climate Commission of the World Meteorological Organization, the Global Climate Research Program, the Climate Change and Predictability Program Climate Change Detection group, and the Expert Team on Climate Change Detection and Indices; these indices were formulated by the Monitoring and Indicators Panel. The indices are divided into four types^42,43^. The relative indices were the number of cool nights, cool days, warm nights, and warm days, which were identified using the relative threshold method. The absolute indices were obtained using a fixed threshold: the number of frost days, summer days, ice days, and tropical nights. The category of “other” indices included the growing season length, warm and cold spell durations, and diurnal temperature range (calculated on a monthly basis). The last category was the extremal indices, which were the maximum and minimum temperatures for the warmest days, warmest nights, coolest days, and coolest nights^18^. We focused on temporal changes and the spatial distributions of those changes for the 16 parameters that described extreme temperatures. These parameters have been extensively studied by international scholars who study extreme climate events^44–49^.

## Methods

We used Mann-Kendall test method to detect significant trends in the rate of change for the extreme-temperature indices at the 45 meteorological stations for regional average values. As a method of nonparametric trend test, it is widely used in the time series trend analysis of hydro-meteorological. The Mann-Kendall test does not require the sample to follow a certain distribution, nor is it subject to the interference of a few abnormal values^50,51^, Thus, it is suitable for the trend assessment of non-normally distributed data. The null hypothesis H_0_ for these tests is that there is no trend in the series. The three alternative hypotheses are that there is a negative, non-null, or positive trend. The Mann-Kendall tests are based on the calculation of Kendall’s tau measure of association between two samples, which is itself based on the ranks with the samples. The computations assume that the observations are independent.

The original Mann–Kendall test statistic (S) is given by

1 $$\begin{array}{rcl}{\rm{S}} & = & {\sum }_{k=1}^{n-1}\,{\sum }_{j=k+1}^{n}Sgn({x}_{j}-{x}_{k})\\ Sgn({x}_{j}-{x}_{k}) & = & 1,{\rm{if}}\,{x}_{j}-{x}_{k} > 0\\ Sgn({x}_{j}-{x}_{k}) & = & 0,{\rm{if}}\,{x}_{j}-{x}_{k}=0\end{array}$$

2 $$Sgn({x}_{j}-{x}_{k})=-\,1,{\rm{if}}\,{x}_{j}-{x}_{k} < 0$$

Where *n* is the length of time series, *x*_*j*_ and *x*_*k*_ represents the sequential data values, respectively.

S is expected to accord with normal distribution with a mean of zero, thus the variance of S could be calculated as follows:

3 $${Var}(S)=\frac{n(n\,-\,1)(2n\,+\,5)}{18}$$

In addition, Z is used to indicate the trend’s significance level, the formulas for calculating Z is as follows:

4 $$\begin{array}{rcl}Z & = & \frac{S-1}{\sqrt{Var(S)}},\,{\rm{if}}\,S > {0}\\ Z & = & {0},\,{\rm{if}}\,S={0}\\ Z & = & \frac{S+1}{\sqrt{Var(S)}},\,{\rm{if}}\,S < {0}\end{array}$$

Thus, when performing a two-sided significance test, the null hypothesis H_0_ would be rejected at significance level of p if |Z| ≥ Z_(1−p/2)_. A positive value of Z represents an upward trend, while a negative value indicates a downward trend in the data. When the absolute value of Z is greater than or equal to 1.28, 1, 64, and 2.32, they pass the significance test of 90%, 95%, and 99%, respectively. Taking into account the effect of autocorrelations in climatic time series, a modified Mann-Kendall trend test was adopted to detect change trends and assess its significance, and Var(S) was improved as follows:

5 $$\mathrm{Var}\ast ({\rm{S}})=\frac{n(n\,-\,1)(2n\,+\,5)}{18}\times \frac{n}{{n}_{s}^{\ast }}$$

6 $$\frac{n}{{n}_{s}^{\ast }}=1+\frac{2}{n(n-1)(n-2)}\times {\sum }_{i=1}^{n-1}(n-i)(n-i-1)(n-i-2){\rho }_{S}(i)$$

where *n* is the number of time series, ${\rho }_{S}(i)$ is the autocorrelation function of the ranks of the observations, and $\frac{n}{{n}_{s}^{\ast }}$ represents a correlation due to the autocorrelations in time series^52^.

We used correlation analysis to detect significant relationships between pairs of indices. We also used one-way ANOVA to detect significant differences in the values of an index between different regions, and when the result was significant, we used least-significant-difference (LSD) tests to identify significant differences between pairs of regions. We displayed the temporal and spatial trends in the extreme indices using version 12.5 of SigmaPlot (https://systatsoftware.com/) and version 10.3 of ArcGIS (http://www.esri.com/). We conducted the statistical tests using version 17.0 of SPSS (https://www.ibm.com/analytics/cn/zh/technology/spss/).

### Data availability

The datasets analyzed during the current study are available from the corresponding author on request.

## Electronic supplementary material

Supplementary Table S1, Supplementary Table S2
