# Supplementary material for: Temporal and spatial variation of extreme temperatures in an agro-pastoral ecotone of northern China from 1960 to 2016
Source: Sci Rep. 2018 Jun 8;8:8787. doi: 10.1038/s41598-018-27066-0 (PMC5993746; doi:10.1038/s41598-018-27066-0)
Supplement: Supplementary file 1 — Supplementary Table S1, Supplementary Table S2 [file 41598_2018_27066_MOESM1_ESM.docx]

Temporal and spatial variation of extreme temperatures in an agro-pastoral ecotone of northern China from 1960 to 2016

Xuyang WANG^1,2^, Yuqiang LI^1,3^ *, Yinping CHEN^4^, Jie LIAN^1,3^, Yongqing LUO^1,3^, Yayi NIU^1,2^, Xiangwen GONG^1,2^, Peidong YU^4^

^1^Northwest Institute of Eco-Environment and Resources, Chinese Academy of Sciences, Lanzhou, 730000, China

^2^University of Chinese Academy of Sciences, Beijing, 100049, China

^3^Naiman Desertification Research Station, Northwest Institute of Eco-Environment and Resources,

Chinese Academy of Sciences, Tongliao, 028300, China

^4^School of Environmental and Municipal Engineering, Lanzhou Jiaotong University, Lanzhou, 730070, China

^*^Corresponding author. E-mail: liyq@lzb.ac.cn

Supplementary Table [S1](http://S1/)**.** Definitions of the temperature and extreme-temperature indices.

| Category | ID | Indicator name | Definitions | Units |
| --- | --- | --- | --- | --- |
| Relative indices | TN10 | Cool nights | Percentage of days when the daily minimum temperature (TN) < 10th percentile | Days |
|  | TX10 | Cool days | Percentage of days when the daily maximum temperature (TX) < 10th percentile | Days |
|  | TN90 | Warm nights | Percentage of days when TN > 90th percentile | Days |
|  | TX90 | Warm days | Percentage of days when TX > 90th percentile | Days |
| Absolute indices | FD | Frost days | Annual number of days when TN <0ºC | Days |
|  | SU | Summer days | Annual number of days when TX > 25ºC | Days |
|  | ID | Ice days | Annual number of days when TX < 0ºC | Days |
|  | TR | Tropical nights | Annual number of days when TN > 20ºC | Days |
| Duration indices | GSL | Growing season length | Annual number of days between the first group of at least 6 days with TG > 5ºC and the first group of 6 days after 1 July (January 1 in SH) with TG < 5ºC | Days |
|  | WSDI | Warm spell duration indicator | Annual number of days with at least 6 consecutive days when TX > 90th percentile | Days |
|  | CSDI | Cold spell duration indicator | Annual number of days with at least 6 consecutive days when TN < 10th percentile | Days |
|  | DTR | Diurnal temperature range | Monthly mean difference between TX and TN | ºC |
| Extremal indices | TXx | Max *T*_max_ | Monthly maximum value of daily maximum temperature | ºC |
|  | TNx | Max *T*_min_ | Monthly maximum value of daily minimum temperature | ºC |
|  | TXn | Min *T*_max_ | Monthly minimum value of daily maximum temperature | ºC |
|  | TNn | Min *T*_min_ | Monthly minimum value of daily minimum temperature | ºC |

Supplementary Table [2](http://S1/)**.** List of meteorological stations in the study area, including the regions to which the stations belong, the station name, latitude, longitude, and elevation.

| Regions | Station name | Latitude (°N) | Longitude (°E) | Elevation (m asl) |
| --- | --- | --- | --- | --- |
| Western Zone | Siziwangqi | 41.53 | 111.68 | 1490.1 |
|  | Huade | 41.9 | 114 | 1482.7 |
|  | Huhehot | 40.82 | 111.68 | 1063 |
|  | Youyu | 40 | 112.45 | 1345.8 |
|  | Jining | 41.03 | 113.07 | 1419.3 |
|  | Datong | 40.1 | 113.33 | 1067.2 |
|  | Dongsheng | 39.83 | 109.98 | 1461.9 |
|  | Hequ | 39.38 | 111.15 | 861.5 |
|  | Yulin | 38.27 | 109.78 | 1157 |
|  | Wuzhai | 38.92 | 111.82 | 1401 |
|  | Yanchi | 37.8 | 107.38 | 1349.3 |
|  | Dingbian | 37.58 | 107.58 | 1360.3 |
|  | Hengshan | 37.93 | 109.23 | 1111 |
|  | Huan county | 36.58 | 107.3 | 1255.6 |
| Central Zone | Zhangbei | 41.15 | 114.7 | 1393.3 |
|  | Weixian | 39.83 | 114.57 | 909.5 |
|  | Duolun | 42.18 | 116.47 | 1245.4 |
|  | Fengning | 41.22 | 116.63 | 661.2 |
|  | Weichang | 41.93 | 117.75 | 842.8 |
|  | Zhangjiakou | 40.78 | 114.88 | 724.2 |
|  | Huailai | 40.4 | 115.5 | 536.8 |
| Eastern Zone | Hailaer | 49.22 | 119.75 | 610.2 |
|  | Xinbaerhuzuoqi | 48.22 | 118.27 | 642 |
|  | Zhalantun | 48 | 122.73 | 306.5 |
|  | Aershan | 47.17 | 119.93 | 997.2 |
|  | Suolun | 46.6 | 121.22 | 499.7 |
|  | Wulanhaote | 46.08 | 122.05 | 274.7 |
|  | Tailai | 46.4 | 123.42 | 149.5 |
|  | Anda | 46.38 | 125.32 | 149.3 |
|  | Baicheng | 45.63 | 122.83 | 155.3 |
|  | Qianan | 45 | 124.02 | 146.3 |
|  | Qianguoerluosi | 45.08 | 124.87 | 136.2 |
|  | Zhaluteqi | 44.57 | 120.9 | 265 |
|  | Tongyu | 44.78 | 123.07 | 149.5 |
|  | Changling | 44.25 | 123.97 | 188.9 |
|  | Linxi | 43.6 | 118.07 | 799.5 |
|  | Kailu | 43.6 | 121.28 | 241 |
|  | Tongliao | 43.6 | 122.27 | 178.7 |
|  | Shuangliao | 43.5 | 123.53 | 114.9 |
|  | Wenniuteqi | 42.93 | 119.02 | 634.3 |
|  | Chifeng | 42.27 | 118.93 | 568 |
|  | Baoguotu | 42.33 | 120.7 | 400.5 |
|  | Zhangwu | 42.42 | 122.53 | 79.4 |
|  | Fuxin | 42.08 | 121.72 | 167.8 |
|  | Yebaishou | 41.38 | 119.7 | 422 |
